# Effects of Electronic Cigarette Use on Cardiovascular-Disease-Related Inflammatory Biomarkers in Smokers with HIV in a Switching Study in the United States

**DOI:** 10.3390/pharma2010010

**Published:** 2023-03-21

**Authors:** Patricia A. Cioe, William V. Lechner, Jennifer W. Tidey, Christopher W. Kahler

**Affiliations:** 1 Center for Alcohol and Addiction Studies, Department of Behavioral and Social Sciences, School of Public Health, Brown University, Providence, RI 02912, USA; 2 Department of Psychological Sciences, Kent State University, Kent, OH 44240, USA

**Keywords:** HIV, tobacco, electronic cigarettes, inflammation, biomarkers

## Abstract

People with HIV (PWH) experience higher rates of cardiovascular events (CVEs) compared with the general population. A substantial body of evidence supports that select biomarkers of inflammation (soluble CD14 [sCD14], soluble CD163 [sCD163], highly sensitive C-reactive protein [hs-CRP], interleukin-6 [IL-6]) and coagulation (D-dimer) are elevated in PWH and related to increased rates of CVEs. Our previous work showed that smoking compared with nonsmoking was associated with significantly elevated sCD14, a biomarker of monocyte activation. We aimed to explore the effect of electronic cigarette (EC) provision on inflammatory biomarkers in PWH who smoked daily and then switched to an EC. Nineteen PWH were enrolled in a pilot study in which an EC and e-liquid were provided weekly for 8 weeks. Blood specimens for inflammatory biomarker analysis were obtained at baseline (BL) and at week 8. Biomarker levels were high at BL and did not differ significantly at week 8. There were small nonsignificant reductions in sCD163 and CRP levels. Nonsignificant increases in IL-6, D-dimer, and sCD14 levels were also noted. Use of ECs for 8 weeks does not appear to significantly increase or decrease inflammatory biomarker levels in SWH. Further research with larger samples and a control group is needed.

## 1. Introduction

People with HIV (PWH) experience higher rates of cardiovascular events (CVEs), including myocardial infarction, compared with the general population [1]. A substantial body of evidence supports that select biomarkers of inflammation (including soluble CD14 [sCD14], soluble CD163 [sCD163], highly sensitive C-reactive protein [hs-CRP], interleukin-6 [IL-6]) and coagulation (D-dimer) are elevated in PWH [2–4] and are related to increased rates of both CVEs and chronic lung disease [5]. These markers remain elevated even in PWH who are effectively treated with ART (ART) and are virologically suppressed [6]. Interventions to reduce levels of inflammatory markers in PWH are limited. While the Reprieve trial is examining the use of pitavastatin to reduce pro-inflammatory responses that may be drivers of CVEs in PWH [7], behavioral strategies to reduce inflammation in PWH (such as reduced cigarette smoking) have not been examined. Importantly, persistent inflammation has been implicated as a contributor to excess mortality risk for PWH compared to those without HIV infection [2,8]. Persistent viremia in PWH has been associated with elevated levels of IL-6 and D-dimer, conferring a 2–3-fold increase in risk for CVEs in PWH [9]. Highly sensitive C-reactive protein (hs-CRP) has been associated with a two-fold increase in the rate of CVEs, independent of other CVD risk factors [10]. Finally, elevated sCD163 has been associated with chronic lung disease in PWH independent of other risk factors [5].

Increased levels of inflammatory biomarkers, coagulation markers, and markers of monocyte activation [11,12] have been associated with cigarette smoking in both PWH and smokers in the general population. Using the Study to Understand the Natural history of HIV and AIDS in the era of effective therapy (SUN Study) [13] dataset, Cioe and colleagues found that among 700 HIV-positive participants, smoking (compared with nonsmoking) was significantly associated with elevated sCD14 [11]. This association was independent of age, CD4 T-cell count, and HIV viral load. Smokers also had numerically higher levels of D-dimer (a marker of coagulation) compared with non-smokers, although this difference was not statistically significant [11]. A more recent study in smokers with HIV demonstrated that cigarettes per day (CPD), number of years smoked, and number of smoking pack years were positively associated with concentrations of the hs-CRP biomarker [12]. A study of smokers in the general population found that current smoking was associated with higher sCD14 biomarker levels in lavage fluid from the smokers’ bronchial alveolar space. They hypothesized that smoke from cigarettes activates sCD14 production by the epithelial cells in the airways, leading to higher levels of inflammation in smokers [14]. Cigarette smoke contains numerous harmful substances that cause endothelial dysfunction and inflammation [15], so it is not surprising that these associations have been reported in smokers with and without HIV.

Although the literature on the effect of smoking cessation or reduction on biomarkers of inflammation among PWH is somewhat limited, biochemically verified smoking cessation in the general population was demonstrated to significantly diminish D-dimer levels at both 6 and 12 months post-cessation [16]. In a second study [17], investigators examined markers of inflammation (hs-CRP), immune activation (sCD14 and sCD163), and coagulation (D-dimer) in HIV-infected and uninfected never, former, and current smokers. Smoking was found to be independently associated with higher hs-CRP levels and lower sCD163 levels, and was associated with higher sCD14 and D-dimer levels at a trend level. No evidence of a differential effect of smoking by HIV status was found [17], and the investigators concluded that cigarette smoking was significantly and independently associated with the markers of inflammation and immune activation. Smoking status, not HIV status, was the differentiating factor in biomarker levels, with former smokers having biomarker levels similar to nonsmokers, suggesting that smoking cessation is beneficial and may be associated with reduced inflammation and biomarker levels.

PWH smoke at much higher rates compared to the general population with reported prevalence rates of approximately 40–50%, and unfortunately, many are unable or unwilling to quit smoking [18–20]. Thus, smoking harm reduction approaches have been suggested to attempt to reduce the harmful health effects of smoking in this vulnerable population. Switching from combustible cigarettes (CCs) to electronic cigarettes (ECs) may represent one form of smoking harm reduction [21]. Several studies have reported that smokers who switch from CCs to ECs report lower levels of CC use, fewer respiratory symptoms, such as wheezing and mucus production [22,23], and reduced rates of exacerbation of chronic obstructive pulmonary disease [24]. While the behavioral and health effects of switching from CCs to ECs in PWH have been reported [22,25], no studies to date have reported the effect of switching from CCs to ECs on biomarkers of inflammation and coagulation in PWH. The purpose of this secondary data analysis was to examine change over time in five cardiovascular-disease-related biomarkers of inflammation (hs-CRP; IL-6; sCD14; sCD163) and coagulation (D-dimer) in smokers with HIV who switched from CCs to ECs for an 8-week period.

## 2. Results

Data were collected from July to December 2018. The baseline demographic and clinical characteristics of the sample are shown in Table 1. All participants were on ART and 17 (89.5%) had an undetectable HIV viral load. During the 8-week intervention period, mean CPD was reduced by more than 80%, from 15.1 9.6 (M SD) at baseline to 1.79 ± 2.2 at week 8. The mean number of EC cartridges used ± per day±by participants over the 8-week period of EC provision was 0.46 (SD = 0.34, range = 0.10–1.24 cartridges daily). Most participants reported using the EC daily. The mean number of days when the EC was not used during the 8-week distribution period was 7.6 days (SD = 10.3), with 11 of the 19 participants (57.8%) reporting EC use daily on 54–56 days during the 8-week period. CO levels decreased significantly from 15.7 ± 7.6 ppm at baseline to 6.7 ppm (M; SD 5.6) at week 8, indicating excellent EC compliance.

Inflammatory biomarker levels were high at baseline and did not differ significantly at week 8 (Table 2). There were, however, small but nonsignificant reductions in mean sCD163 levels from baseline [M = 883.6, SD = 395.2] to week 8 [M = 791.4, SD = 357.7], t(1.14), *p* = 0.281, d = 0.26, and decreases in mean hs-CRP levels from baseline [M = 3799.5, SD = 2023.6] to week 8 [M = 3489.1, SD = 1876.1], t(0.642), *p* = 0.529, d = 0.15. Non-significant increases in IL-6, D-dimer, and sCD14 were also noted: (IL-6: BL (M = 3.16, SD = 2.03), week 8 (M = 3.56, SD = 3.73), t(−0.422), *p* = 0.678, d = 0.099; D-dimer: BL (M = 513.6, SD = 327.6), week 8 (M = 578.9, SD = 237.7), t(−1.125), *p* = 0.276, d = 0.27; sCD14: BL (M = 1,861,198.7, SD = 660,532.9), week 8 (M = 2,197,481.4, SD = 786,839.9), t(−1.08), *p* = 0.293, d = 0.26).

## 3. Discussion

This study evaluated changes in levels of serum biomarkers of inflammation and coagulation in smokers living with HIV who were encouraged to switch from CCs to ECs for 8 weeks. We did not find significant changes in any of the five serum inflammatory biomarkers that we examined. We hypothesized that switching from CCs to ECs would result in a reduction in inflammatory biomarkers, based on previous work [11]. While our previous work showed that current smoking compared with nonsmoking was associated with significantly elevated sCD14, there was no observable reduction in sCD14 in smokers who switched from CCs to ECs in this sample of 19 participants. There were also no reductions seen in D-dimer or IL-6 levels in our participants.

The five biomarkers selected for study have been found to be associated with higher rates of CVEs in PWH in previous studies, as described earlier. While there were small but nonsignificant reductions in sCD163 and hs-CRP seen in our sample, we also observed small, non-significant increases in IL-6, D-dimer, and sCD14. The clinical significance of these changes is unclear. While the reductions in two biomarkers may be promising as elevations in these biomarkers have been associated with higher rates of CVEs in PWH, further study in larger samples over longer periods of time is clearly needed. Previous studies have shown that smokers had increased hs-CRP, irrespective of HIV status, and increased plasma cotinine concentrations were associated with increased hs-CRP [26], so we were encouraged to see a signal indicating reductions in two of the examined biomarkers in smokers who switched to ECs. While the clinical significance of these findings remains unclear, further investigation of the potential of tobacco harm reduction for smokers who are unable or unwilling to quit is warranted. Importantly, an 8-week period of switching from CCs to ECs does not appear to significantly increase inflammatory biomarker levels in smokers with HIV.

This study has several strengths. To our knowledge, it is the first study to examine change in biomarkers of inflammation and coagulation in smokers with HIV who switch from CCs to ECs. Secondly, we used an EC and e-liquid combination that is commercially available and has been shown to deliver physiologically active nicotine doses [27,28]. A major limitation of this study is the small sample size, limiting our ability to see significant changes in biomarker levels. Additionally, the 8-week duration of EC use may have limited our findings, since the optimal period required for change in biomarker levels, related to smoking behaviors, is not known. Finally, the lack of a control group is a significant limitation. Further study with larger samples, longer duration of EC use, perhaps several points of biomarker measurement, and a control group of smokers who continue to smoke their usual brand is clearly needed. Finally, we were not able to examine change in those smokers who made a complete switch to an EC, compared to those who became dual users (ECs and CCs), which limits our ability to fully examine the effect of EC provision on tobacco-related harm in this population. Goniewicz and colleagues (2018) have shown that exclusive EC use results in lower biomarker concentrations of nicotine and other tobacco-related toxicants compared with CC smoking [29], while Stokes and colleagues showed that biomarkers of inflammation (hs-CRP, IL-6) are lower in exclusive electronic cigarette users, compared to smokers and dual users [30]. While seven participants stopped smoking CCs by week 8, most of our participants continued to smoke at least 1–2 CPD, which may have continued to contribute to inflammation. A larger sample would allow us to examine differences in biomarkers of inflammation and coagulation in all three groups (CC smokers, dual CC/EC users, and complete EC switchers) as well.

## 4. Methods

This paper describes a secondary data analysis. The primary study examined the feasibility and acceptability of EC provision for eight weeks to smokers with HIV who were not ready or not willing to quit smoking. The findings of the primary study have been previously described [22]. In this analysis, we examine whether any change in biomarkers of inflammation and coagulation occurred in smokers who switched from CCs to an EC.

Smokers with HIV were recruited from a large HIV clinic in the northeast United States. Flyers were hung on study bulletin boards in the clinic and patients who were interested called the study center for screening. Eligibility criteria included over 18 years old; smoked > 5 CPD for longer than one year; and exhaled breath CO level > 5 ppm (parts per million) at baseline. Smokers were excluded if they had been hospitalized in the past month, had unstable angina or hypertension, had a substance use disorder other than nicotine, or were pregnant. Smokers were also excluded if they had plans to quit smoking in the next 30 days, were using smoking cessation therapy, or were using electronic cigarettes more than 2 days per week.

Twenty smokers living with HIV were enrolled in the open pilot study; one participant dropped out immediately after enrollment due to a new job and inability to attend study visits. An EC and e-liquid were provided weekly for 8 weeks to the remaining 19 participants. Participants were given two ce6 eGo-T electronic cigarette devices (second generation pen-style), which had a 3.3 volt, 1100 mAh battery with 6–10 1.5 Ohm, dual coil XL, 510-style cartomizer (SmokTech; Shenzhen, China). The electronic cigarette liquid had 18 mg/mL nicotine per milliliter with a 30/70 PG/VG ratio. Participants were provided with enough e-liquid each week so that they did not run out between visits. Three flavor choices were available (Tobacco Row (tobacco), Arctic Blast (menthol), and Mardi Gras (fruit)), and participants could select various flavors each week. E-liquid was manufactured by Avail Vapor (Richmond, VA) and was tested to ensure the concentration of the nicotine was accurate. Participants were encouraged to use the EC daily, whenever they would normally smoke a cigarette. Serum biomarkers of inflammation, coagulation, cigarette use, and carbon monoxide (CO) exposure were obtained at baseline (BL; prior to EC use) and at week 8 (end of e-liquid provision period). All study procedures were approved by the Brown University Institutional Review Board (Protocol #1804002047) and all participants signed a written informed consent form to participate. Participants were compensated up to USD 260 for sessions completed.

### 4.1. Measures

#### 4.1.1. Demographics and Clinical Characteristics

Age, sex assigned at birth, education level, employment status, race/ethnicity, and number of years living with HIV were obtained by self-report at baseline. Number of CPD, number of years smoked, and type of cigarette (menthol/regular) were also obtained by self-report. The most recent CD4 Tcell count and the HIV viral load were obtained from the medical record.

#### 4.1.2. Exhaled Carbon Monoxide (CO) Level

Participants were asked to provide an exhaled breath sample for CO measurement at the baseline and week 8 visits. A Bedfont Smokerlyzer ED50 meter (Bedfont Scientific, Haddonfield, NJ, USA) was used for all CO measurements. Carbon monoxide is a product of cigarette combustion, and CO levels < 5 ppm are indicative of abstinence from CC use [31].

#### 4.1.3. Serum Biomarkers

The following biomarkers of inflammation and coagulation were obtained: sCD14, a biomarker of monocyte activation that is associated with tobacco use [11], atherosclerosis [32], and mortality in PWH; sCD163, a biomarker of monocyte/macrophage activation, which is independently associated with chronic lung disease in PWH; hs-CRP, an acute phase reactant, which has been linked to elevated cardiovascular risk [33,34]; D-dimer, a marker of coagulation, which has been shown to be elevated in PWH and has been associated with cardiac events [2,3,35,36]; and IL-6, a biomarker of inflammation, which has been associated with increased rates of CVEs [36].

#### 4.1.4. Biomarker Collection and Analysis

Venous blood samples were collected from participants at baseline (prior to EC use) and 8 weeks (at the end of the EC provision period). At each time point, two 10 mL plasma samples were collected into BD Vacutainer tubes and placed on ice. Samples were centrifuged at 1700× *g* for 15 min. Using a p1000 pipette, a serum layer was aspirated from the centrifuged tube and stored in endotoxin-free cryovials at −80 °C. Enzyme-linked immunosorbent assays (ELISA) were used to quantify D-dimer (Cat no, ab196269; abcam, Cambridge, MA, USA), sCD14 (Cat no. DC140; R&D Systems, Minneapolis, MN, USA), sCD163 (Cat no. DC1630; R&D Systems, Minneapolis, MN, USA), hs-CRP (Cat no. DCRP00), and IL-6 (Cat no, HS600C) following manufacturer instructions. For each sample, duplicate ELISA tests were performed using a GloMax^®^ Discover Microplate Reader (Promega, Madison, WI, USA).

### 4.2. Data Analysis

Analyses were performed using SPSS version 24. Paired samples t-tests were con ducted, and Cohen’s D (mean difference/SD of the difference) was calculated, to examine whether markers of inflammation (IL-6, D-dimer, hs-CRP, sCD14, and sCD163) improved significantly from BL to 8 weeks. Examination of skewness and kurtosis of changes in biomarkers did not indicate substantial departure from normality [37]. One outlier (range = 3rd quartile + 3 × interquartile range, 1st quartile − 3 × interquartile) was identified for the IL-6 marker; significance did not change with or without the outlier included in analyses. Data presented in Section 2 include the outlier value.

## 5. Conclusions

In summary, in this small EC switch study of smokers with HIV, we found small, nonsignificant reductions in some key biomarkers of inflammation that have been associated with increased morbidity and mortality in PWH. This is an initial step to examine whether ECs may be beneficial as a tobacco harm reduction strategy in smokers with HIV. Further study is warranted, as tobacco harm reduction for PWH is a priority since some smokers are ambivalent about quitting smoking [38]. Reducing the harmful health effects of cigarette smoking by encouraging a switch to ECs may be important, either as a final step in tobacco harm reduction or as an intermediate step toward complete smoking cessation.

## Figures and Tables

**Table 1.** Sample characteristics at baseline (*n* = 19).

Variable	Mean (SD)	Range
Age	53.6 (8.7)	32–66
Carbon Monoxide, exhaled (ppm)	15.7 (7.7)	6–32
CPD	15.1 (9.6)	6–40
Number of Years Smoked	32.9 (12.4)	7–50
CD4 T-cell count	765.9 (363.8)	307–1581
Years Living with HIV	21.1 (10.2)	4–38
Variable		*n* (%)
Type of Cigarette Smoked		
Menthol		12 (63%)
Race/Ethnicity		
Hispanic or Latino		3 (16)
Non-Hispanic White		10 (53)
Non-Hispanic Black or African American		5 (26)
Non-Hispanic Multi-Racial		1 (5)
Employment Status		
Employed, part/full-time		3 (16)
Unemployed		5 (26)
Disabled		11 (58)
Education		
Less than High School		8 (42)
High School/Some College		9 (47)
College Graduate or Higher		2 (11)
Sex Assigned at Birth		
Female		6 (32)
Male		13 (68)

**Table 2.** Mean (SD) biomarker for plasma samples at baseline, week 8, and mean difference.

ELISA Assay	Baseline Mean (SD)	Week 8 Mean (SD)	BL and W8 Mean Difference	95% CI	*t*	*p*-Value
sCD14	1,861,198.7 (660,532.9)	2,197,481.4 (786,839.9)	336,282.72	(990,665.04, 318,099.59)	−1.08	0.29
sCD163	883.6 (395.2)	791.4 (357.7)	−92.23	(−82.38, 266.84)	1.14	0.28
hs-CRP	3799.5 (2023.6)	3489.1 (1876.1)	−310.46	(−709.79, 1330.71)	0.64	0.53
IL-6	3.16 (2.03)	3.56 (3.73)	0.41	(−2.44,1.62)	−0.42	0.68
D-Dimer	513.6 (327.6)	578.9 (237.7)	65.34	(−187.83, 57.16)	−1.13	0.28

## Data Availability

Data may be available by written request to the Corresponding Author.
